# Real-World Approach for Molecular Analysis of Acquired EGFR Tyrosine Kinase Inhibitor Resistance Mechanisms in NSCLC

**DOI:** 10.1016/j.jtocrr.2021.100252

**Published:** 2021-11-01

**Authors:** Liesbeth M. Hondelink, Merel Jebbink, Jan H. von der Thüsen, Danielle Cohen, Hendrikus J. Dubbink, Marthe S. Paats, Anne-Marie C. Dingemans, Adrianus J. de Langen, Mirjam C. Boelens, Egbert F. Smit, Pieter E. Postmus, Tom van Wezel, Kim Monkhorst

**Affiliations:** a Department of Pathology, Leiden University Medical Center (LUMC), Leiden, The Netherlands; b Department of Thoracic Oncology, The Netherlands Cancer Institute (NKI), Amsterdam, The Netherlands; c Department of Pathology, Erasmus Medical Center (EMC), Rotterdam, The Netherlands; d Department of Respiratory Medicine, Erasmus MC Cancer Institute, University Medical Center, Rotterdam, The Netherlands; e Department of Pathology, The Netherlands Cancer Institute (NKI), Amsterdam, The Netherlands; f Department of Pulmonology, Leiden University Medical Center (LUMC), Leiden, The Netherlands

**Keywords:** Non–small cell lung cancer, Molecular diagnostics, Tyrosine kinase inhibitors, Acquired resistance

## Abstract

**Introduction:**

With the approval of first-line osimertinib treatment in stage IV EGFR-mutated NSCLC, detection of resistance mechanisms will become increasingly important—and complex. Clear guidelines for analyses of these resistance mechanisms are currently lacking. Here, we provide our recommendations for optimal molecular diagnostics in the post-EGFR tyrosine kinase inhibitor (TKI) resistance setting.

**Methods:**

We compared molecular workup strategies from three hospitals of 161 first- or second-generation EGFR TKI–treated cases and 159 osimertinib-treated cases. Laboratories used combinations of DNA next-generation sequencing (NGS), RNA NGS, in situ hybridization (ISH), and immunohistochemistry (IHC).

**Results:**

Resistance mechanisms were identified in 72 first-generation TKI cases (51%) and 85 osimertinib cases (57%). RNA NGS, when performed, revealed fusions or exon-skipping events in 4% of early TKI cases and 10% of osimertinib cases. Of the 30 MET and HER2 amplifications, 10 were exclusively detected by ISH or IHC, and not detected by DNA NGS, mostly owing to low tumor cell percentage (<30%) and possibly tumor heterogeneity.

**Conclusions:**

Our real-world data support a method for molecular diagnostics, consisting of a parallel combination of DNA NGS, RNA NGS, MET ISH, and either HER2 ISH or IHC. Combining RNA and DNA isolation into one step limits dropout rates. In case of financial or tissue limitations, a sequential approach is justifiable, in which RNA NGS is only performed in case no resistance mechanisms are identified. Yet, this is suboptimal as—although rare—multiple acquired resistance mechanisms may occur.

## Introduction

Approximately 11% of all lung adenocarcinomas harbor a driver mutation in the EGFR gene.^1^ Most of these EGFR mutations have been targeted with first- and second-generation tyrosine kinase inhibitors (TKIs) for several years, resulting in a substantial improvement of both overall and progression-free survival for these patients.^2^^,^^3^ In 2017, osimertinib, a third-generation TKI, was approved by the Food and Drug Administration and European Medicines Agency as second line^4^ and more recently as first line for the treatment of metastatic EGFR-mutated NSCLC, which further improved survival.

Although targeted treatment with selective TKIs has been found to improve overall survival substantially, all tumors eventually acquire resistance, inevitably resulting in death.^5^ In first- and second-generation TKI resistance (such as erlotinib, gefitinib, afatinib), acquired resistance mechanisms predominantly consist of on-target mutations in EGFR, mainly T790M,^6^, ^7^, ^8^, ^9^ but also D761Y,^10^ L747S,^11^ and T854A point mutations and EGFR amplification.^6^, ^7^, ^8^, ^9^^,^^12^ Off-target resistance mechanisms include mutations in BRAF, PIK3CA, and KRAS, amplifications of HER2 and MET, oncogenic fusions in RET, FGFR3, ROS1, and NTRK, and MET- and EGFR-exon skipping and transformation into SCLC.^6^, ^7^, ^8^, ^9^^,^^13^, ^14^, ^15^, ^16^, ^17^, ^18^ Squamous transformation has so far only been described in case reports after first- and second-generation TKIs.^19^

For osimertinib treatment (both first line and later lines), the most frequent on-target resistance mutation is C797S,^20^, ^21^, ^22^ although G724S, G796, L792, L718, G719, L844, and V834 have also been reported. In contrast to first- and second-generation TKIs, off-target mechanisms occur more frequently and are more heterogeneous. Off-target resistance mechanisms after osimertinib include not only all resistance mechanisms after earlier TKIs but also amplifications of FGFR1 and transformation to a squamous phenotype.^20^^,^^21^^,^^23^, ^24^, ^25^ Off-target resistance mechanisms are more prevalent after osimertinib compared with the first- and second-generation TKI–treated cases.^16^

The volume of patients who are referred to a tertiary referral hospital for EGFR TKI resistance mechanism screening is increasing. This number will likely continue to rise even more in the years to come, owing to improved access to TKIs, sequential use of different TKIs, and adjuvant TKI treatment for earlier stages of NSCLC. Several of these acquired resistance mechanisms are currently or will soon become treatable^24^^,^^26^ through regular reimbursed treatment or in an experimental, off-label, early access, or compassionate-use setting, which makes adequate screening for acquired resistance essential.

Although single-assay screening (with whole-genome sequencing [WGS] or large hybrid capture panel) is the most elegant method of screening owing to its completeness, currently this is not yet feasible in most laboratories worldwide. Small biopsies and cytology specimens still are the mainstay of tissue procurement during EGFR TKI therapy, which limits the potential broad applicability of large panel strategies, for which larger amounts of tumor material are necessary. Moreover, even large hybrid capture panels sometimes miss exon-skipping events, oncogenic fusions, and copy number variation owing to the length of introns, blind spots within the targeted areas, and large deletions, which cannot be captured.

In practice, a consensus on how to screen for these resistance mechanisms is currently lacking. This results in substantial differences between laboratories. This disagreement is largely explained by the broad spectrum of possible acquired resistance mechanisms, with potential co-occurrence, and the broad range of potential screening modalities, each with their own advantages and limitations. Thus, although DNA next-generation sequencing (NGS) panels detect point mutations, deletions, and insertions, they fail to detect most fusions and exon-skipping events and occasionally miss copy number variation as well, especially if the tumor cell percentage is low. In situ hybridization (ISH) or immunohistochemistry (IHC) for fusion targets and amplifications is a single-target assay that constitutes a time- and tissue-consuming challenge. RNA NGS is the preferred technique to detect both exon-skipping events and fusions, including their fusion partners, but current RNA NGS panels do not detect all point mutations, deletions, and insertions that DNA NGS can detect. In conclusion, the multitude of potential resistance mechanisms combined with a multitude of potential techniques to detect them presents to both thoracic pathologists and molecular biologists the complex challenge of choosing the optimal workup for tumor biopsies from patients progressing on EGFR TKI therapy.

This multicenter study therefore aims to provide recommendations on the most efficient and effective resistance analysis after EGFR TKI treatment, by evaluating the existing workflow in a retrospective “real world” cohort analysis that includes 320 routinely acquired resistance biopsy specimens analyzed in three specialized hospitals in The Netherlands. We aimed to address five “challenges” in effective screening after EGFR TKI resistance, which are as follows: somatic mutation detection, fusion detection, amplification detection, tissue scarcity, and comparison to the pretreatment biopsy. By addressing these challenges step by step, we will propose a workup that takes into account the added value and effectivity of each test modality and is specifically tailored to deal with specific EGFR TKI resistance issues, such as (non)mutual exclusivity and tissue scarcity.

## Material and Methods

### Study Setup

We included 320 EGFR-mutated NSCLC biopsy specimens from 248 patients (317 adenocarcinomas and three squamous cell carcinomas) from three hospitals in The Netherlands, which were submitted to the pathology department for EGFR TKI resistance analysis between January 2018 and February 2020. The biopsy specimens were included in the early TKI group when the patient had acquired resistance to a first- or second-generation TKI, such as erlotinib, gefitinib, and afatinib. The biopsy specimens were included in the osimertinib group when the patient had acquired resistance to osimertinib.

Tumors (n = 3) that originally presented with neuroendocrine differentiation were excluded, owing to the morphologic and molecular differences with NSCLC. Cytology and non-cytology materials were both included. Patients receiving first-line TKI treatment were included and later therapy lines. Patients harboring tumors that became resistant to multiple TKI lines were included twice: once in the early TKI group after the first resistance to the first- or second-generation TKI and once in the osimertinib group after resistance to osimertinib. Some patients were treated with first-line osimertinib, but most received multiple TKI lines (Supplementary Fig. 1). In addition, in some patients, the first resistance biopsy specimen did not yield a resistance mechanism, so it was repeated. Those biopsy specimens were included as well. These “double inclusions” occurred in 60 patients and reflects the “real world” TKI resistance setting, in which pathologists are required to perform resistance analysis multiple times for the same patient.

The laboratories performed RNA NGS, MET ISH, DNA NGS, HER2 IHC, or HER2 ISH to varying degrees. The laboratories were all NEN-EN-ISO 15189 accredited, which includes regular evaluations, audits, and quality checks. Due to the retrospective, anonymized nature of this study, informed consent was not required.

### DNA NGS

DNA NGS was performed with laboratory-specific customized oncogene panels that cover hotspots in relevant genes, including EGFR, MET, HER2, KRAS, BRAF, PIK3CA, FGFR1, FGFR2, and FGFR3, and several other mutations. Copy number analysis was performed with the DNA NGS data by locally validated pipelines. Details on all other genes included in the customized NGS panels and copy number analysis pipelines are available in the Supplementary Methods. The panels vary slightly, but relevant resistance mechanisms, which are recited in the Introduction section, are covered in each panel.

### RNA NGS

All laboratories used anchored multiplex polymerase chain reaction–based NGS (RNA NGS) technology from Archer DX. Either the FusionPlex Comprehensive Thyroid and Lung Panel or the FusionPlex Lung Panel was used. Reads were analyzed with vendor-supplied software on an IonTorrent platform. The panels used included fusions and exon-skipping events in ALK, BRAF, EGFR, FGFR1, FGFR2, FGFR3, MET, NRG1, NTRK1, NTRK2, NTRK3, RET, and ROS1. A comprehensive overview of the methods used for RNA NGS is included in the Supplementary Methods.

### RNA and DNA Isolation

All analyses were performed with formalin-fixed, paraffin-embedded (FFPE) tissue, including cell blocks from cytology specimens. DNA and RNA were isolated differently in each laboratory. At the Erasmus Medical Center, DNA was isolated with Chelex or Maxwell, as previously described, whereas RNA was isolated with the Qiagen method. At the Leiden University Medical Center, total nucleic acid was isolated with a Siemens tissue preparation robot and used for both DNA NGS and RNA NGS as previously described.^27^ At the Netherlands Cancer Institute, DNA and RNA were isolated separately with a Qiagen FFPE preparation kit.

If DNA and RNA were isolated separately, DNA was stored at −20°C and RNA at −80°C. If total nucleic acid was isolated, the isolate was stored at −20°C short term and −70°C long term. A more detailed description of the RNA and DNA isolation process is supplied in the Supplementary Methods. Tumor cell percentage was considered “low” if it was below 30%.

### ISH (MET and HER2 ISH)

HER2 ISH was either performed with Ventana Dual ISH and stained on the Ventana Benchmark Ultra or with Dual SISH from Roche Diagnostics. MET ISH was performed with Dual Color MET-Cen7 probe either from Leica Kreatech, Zytolight Spec, or Roche Diagnostics. Additional information regarding the ISH is available in the Supplementary Methods.

### HER2 IHC

Slides were either stained for HER2 with the Dako A0485 antibody on the Dako Omnis immunostainer using Dako EnVision Flex+ in a laboratory developed test with citrate and a 1:100 dilution or stained on the Benchmark Ultra with Ventana 4B5 antibody. A more comprehensive explanation on the protocol for IHC is in the Supplementary Methods.

### Morphologic Examination and Typing

All cases were evaluated by one expert thoracic pathologist per center (DC, KM, JT) and classified according to the 2015 WHO classification. Immunohistochemical staining was used for typing when indicated. In case of suspected morphologic transformation to squamous or small-cell phenotype, this was confirmed by IHC (synaptophysin, CD56, and chromogranin for small-cell, P40 for squamous).

### Molecular Comparison to Pretreatment Biopsy

All molecular profiles of resistance biopsies were compared with the molecular profile of the pretreatment biopsy where possible. We considered a molecular alteration in the resistance biopsy an “acquired resistance mechanism” if (1) the alteration was absent in the pretreatment biopsy and (2) the molecular alteration was considered to be a class 4 or 5 pathogenic mutation, reported to be associated with an acquired EGFR TKI resistance phenotype in previous literature, such as EGFR T790M, KRAS G12C, and BRAF V600E. On the basis of the literature, we assumed that treatment naive, EGFR-mutated tumors do not harbor oncogenic fusions. Owing to this assumption, first-line TKI resistance biopsy specimens could be compared with treatment-naive specimens without pretreatment RNA NGS.

We considered molecular alterations “acquired driver mutations” if (1) the alteration was considered to be a class 4 or 5 pathogenic alteration, but not reported to be associated with an acquired EGFR TKI resistance phenotype, such as TP53, CDKN2A, and CTNNB1, and (2) the alteration was absent in the pretreatment biopsy.

**Figure 1:**
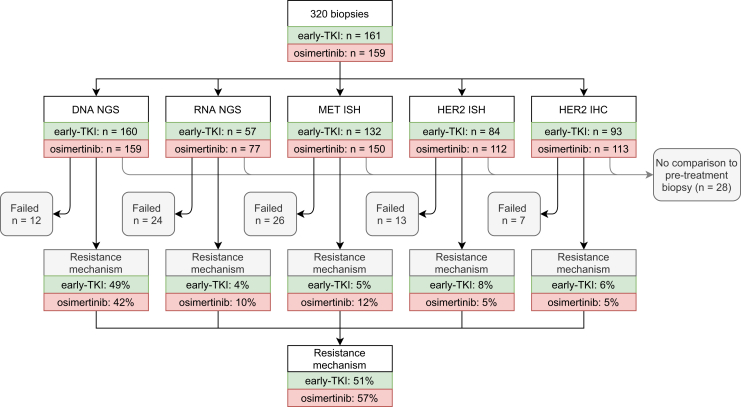
Performed DNA NGS, RNA NGS, ISH, and IHC in this study for each treatment group. Percentages for each test are based on successful analyses, and total percentage of resistance mechanisms (51% and 57%) is based on all attempted analyses that could be compared with the pre-TKI biopsy, including analyses which returned no result owing to insufficient tissue. IHC, immunohistochemistry; ISH, in situ hybridization; NGS, next-generation sequencing; TKI, tyrosine kinase inhibitor.

There were several situations in which molecular comparison of the resistance biopsy and the pretreatment biopsy was suboptimal or impossible, for instance in case of incomplete molecular workup of the pretreatment biopsy owing to scarce material, with liquid biopsy as the only pretreatment material. In the setting of suboptimal comparability of molecular profiles, cases were excluded from the analyses of resistance mechanism prevalence, as illustrated in Figure 1. We used the Alamut, CKB, OncoKB, Franlinn, and Cosmic databases for pathogenicity assessment.

### Amplifications

Amplifications for all genes, except MET and HER2, were considered amplifications if the estimated copy number was 10 or more. For MET and HER2, an estimated copy number between six and 10 was considered “low amplification,” and an estimated copy number of more than 10 “high amplification,” as MET and HER2 amplifications with six to 10 copies can be clinically relevant.^28^^,^^29^

HER2 IHC scoring was performed by a customized scoring system. The percentage of tumor cells with “no staining,” “low intensity staining,” “moderate intensity staining,” and “high intensity staining” was estimated by histopathologic examination. Cases were considered to have a score of 0 if 90% or more tumor cells had no or low-intensity staining. Cases were considered to have a score of 1+ if more than 50% but less than 90% of the tumor cells had low-intensity staining. Cases were considered to have a score of 2+ if more than 50% but less than 90% of the tumor cells had moderate- or high-intensity staining. Cases were considered to have a score of 3+ if 90% or more tumor cells had high-intensity staining. Staining was based on membranous HER2 staining. Cells with incomplete membranous staining were considered positive.

### Smoking

Patients were considered to be never smokers if they did not smoke at least 1 month before the NSCLC diagnosis and had accumulated fewer than two pack-years in their lifetime. Patients were considered to be former smokers if they had stopped smoking more than 1 month before they were first diagnosed with NSCLC and had accumulated two pack-years or more. Patients were considered to be current smokers if they had smoked in the month before being diagnosed with NSCLC, regardless of pack-years.

### Statistics

Statistical analysis was performed using IBM SPSS Statistics software, version 25. OncoPrints were visualized with cBioPortal version 3.5.4 OncoPrinter.^30^^,^^31^

### Ethics

The data were obtained from routine diagnostic reports and anonymized before processing. This study was approved by the institutional review board at the Netherlands Cancer Institute.

## Results

### Specimen Collection

We included 320 biopsy specimens from 248 patients in this study. Characteristics are outlined in Table 1. Most of the patients were of female sex or never smoker (Table 1 and Supplementary Table 1), more frequent than has been described in the treatment-naive advanced-stage NSCLC population in The Netherlands.^32^

**Table 1:** Specimen Characteristics for Each Treatment Group, Registered Per Specimen (N = 320)

Characteristics	Early TKI Group (n = 161)	Osimertinib Group (n = 159)	*p* Value
Sex, n (%)			0.55^a^
Female	105 (65)	109 (69)	
Male	56 (35)	50 (31)	
Age	65 (31–89)	63 (32–86)	0.09^b^
Biopsy site, n (%)			0.73^c^
Primary tumor	58 (36)	53 (33)	
Lymph node	23 (14)	20 (13)	
Distant metastasis	80 (50)	86 (54)	
Tumor type, n (%)			0.12^a^
Adenocarcinoma	161 (100)	156 (98)	
Squamous cell carcinoma	0	3 (2)	
Specimen type, n (%)			0.004^a^
Cytology	64 (40)	39 (25)	
Non-cytology	97 (60)	120 (75)	

TKI, tyrosine kinase inhibitor.

a *p* values were calculated with Fisher’s exact test.

b *p* values were calculated with unpaired *t* test.

c *p* values were calculated with Pearson’s chi-square test.

The early TKI group included significantly more cytology specimens than the osimertinib group (*p* = 0.004, Fisher’s exact test). This is likely due to the more frequent use of endobronchial or endoesophageal ultrasound-guided lymph node aspiration in the referring hospitals. Several patients were included in both the early TKI group and the osimertinib group, reflecting use of second-line osimertinib after resistance to the first- or second-generation TKI. The timeline of this patient group is outlined in Supplementary Figure 1.

### Challenge #1 Somatic Mutation Detection

**Figure 2:**
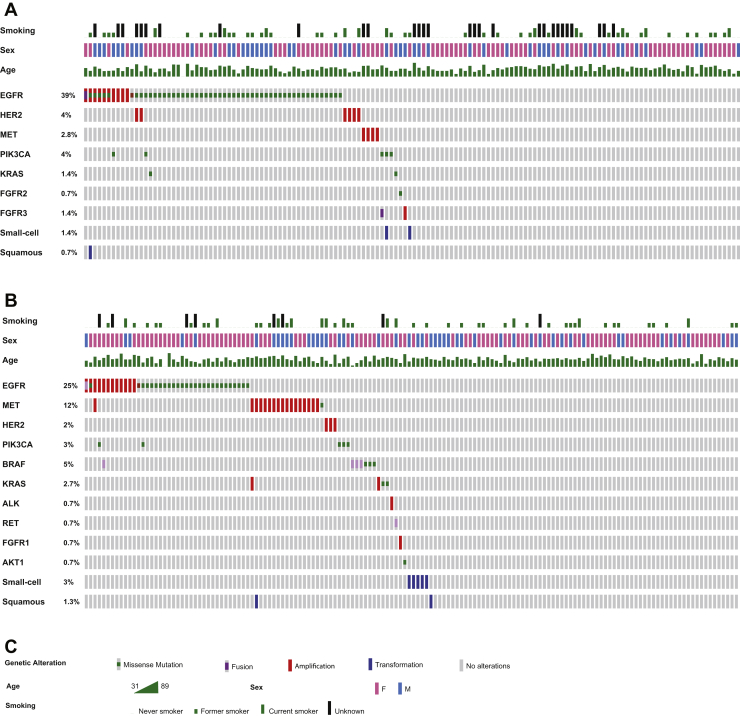
(*A*) OncoPrint for the first- and second-generation TKI resistance cohort (early TKI group). EGFR mutations include the following: A298V, I706T (VUS), K754E, S768I, T790M, C797S, and exon skipping. (*B*) OncoPrint for the third-generation TKI resistance cohort (osimertinib group). EGFR mutations include the following: L62R, A298T, L718Q (VUS), G724S, I744M, G796S, C797S, L972H, and exon skipping. (*C*) Legends. All listed mutations are pathogenic driver mutations, which were not present in the pretreatment biopsy. This includes the listed EGFR mutations: the original EGFR mutation is not included in this figure. F, female; M, male; TKI, tyrosine kinase inhibitor; VUS, variant of unknown significance.

DNA NGS was used to screen for somatic mutations, including point mutations and small deletions and insertions. DNA NGS was performed in 319 of 320 cases and was successful in 307 cases, as outlined in Figure 1. In the early TKI group, DNA NGS detected a resistance mechanism in 66 early TKI cases (49% of successful tests) and in 62 osimertinib cases (42% of successful tests). The identified somatic mutations are summarized in Figure 2*A*–*C* and are often, but not always, mutually exclusive with other resistance mechanisms. We used the definition for “acquired resistance mechanisms” as described in the Material and Methods section.

**Figure 3:**
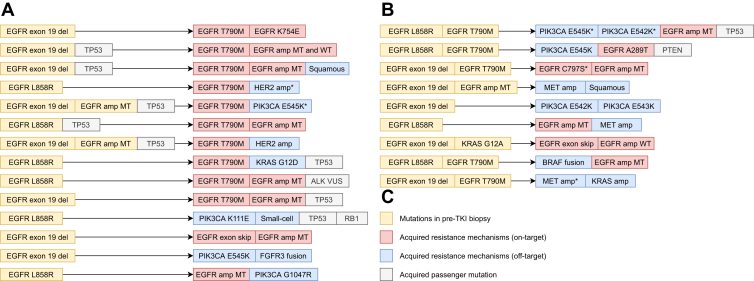
Cases harboring multiple resistance mechanisms. (*A*) First- and second-generation TKI resistance cohort (early TKI group). (*B*) Third-generation TKI resistance cohort (osimertinib group). (*C*) Legend. Mutations marked with ∗ are present in only part of the tumor cells, indicating clonal heterogeneity. amp, amplification; del, deletion; MT, mutant; TKI, tyrosine kinase inhibitor; WT, wild-type.

Mutual exclusivity is outlined in Figure 3*A–C*, where we reveal that in 23 cases overall (7% of all cases), multiple new resistance mechanisms are present in the resistance biopsy. In the early TKI group, multiple resistance mechanisms were detected in 14 cases (9% of all early TKI cases), and in the osimertinib group in nine cases (6% of all osimertinib cases). The prevalence of co-occurring mutations in resistance biopsies is substantial, especially considering not all biopsies underwent RNA NGS and ISH, as found in Figure 1. Nevertheless, when we look closer at which resistance mechanisms co-occur, we observe that it is frequently (in 16 of 23 cases, 70%) PIK3CA or EGFR amplification in concurrence with another mutation. Co-occurrence of “strong” resistance mechanisms, such as T790M, HER2 amp, KRAS, or MET amp, is rare and occurs only in seven cases in this cohort (2%).

Acquired resistance EGFR mutations in the early TKI group include the following: A298V, I706T (variant of unknown significance), K754E, S768I, T790M, C797S, and EGFR-exon skipping (exons 21–27 or exons 2–7). EGFR mutations in the osimertinib group include the following: L62R, A298T, L718Q (variant of unknown significance), G724S, I744M, G796S, C797S, L972H, and EGFR-exon skipping. We conclude that the identified on-target and off-target resistance mechanisms are similar to those identified in the literature, for both treatment groups.

### Challenge #2 Fusion and Exon-Skipping Detection

**Figure 4:**
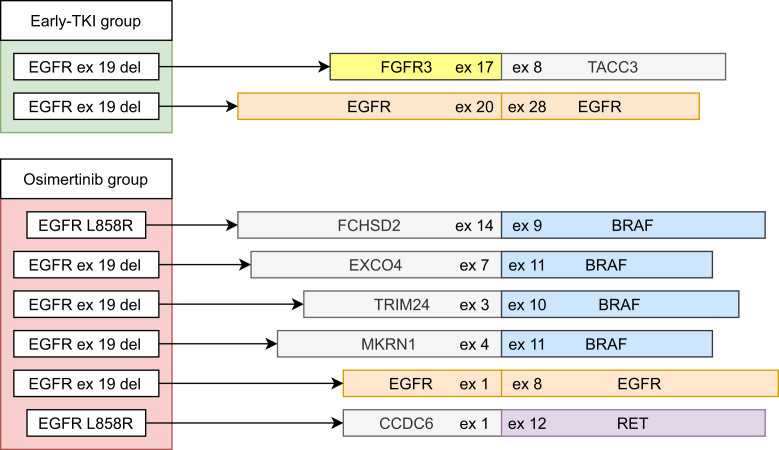
Fusions and exon-skipping events identified in RNA NGS. del, deletion; ex, exon; NGS, next-generation sequencing.

RNA NGS was performed in 134 cases. It was successful in 110 cases (82%), whereas in 24 cases (18%), insufficient RNA was available for the analysis. In eight cases overall (7% of all successful analyses), an exon-skipping or fusion event was found by RNA NGS, all of which are visualized in Figure 4. These events occurred twice in the early TKI group (4% of successfully tested cases) and six times in the osimertinib group (10%, *p* = 0.46, Fisher’s exact test). The identified fusions and exon-skipping events were not mutually exclusive with other resistance mechanisms, as outlined in Figures 2 and 3; instead they co-occurred with other resistance mechanisms in four cases. In the early TKI group, both rearrangements co-occurred with other resistance mechanisms, being PIK3CA mutation and EGFR amplification, respectively. In the osimertinib group, two of six fusions or exon-skipping events overlapped with other mechanism (33%), both with an EGFR amplification. This is in line with the literature, where co-occurrence of a fusion or exon-skipping event with a stronger resistance mechanism, such as BRAF, KRAS, EGFR T790M, or MET amplification, has not been found often.

Several of the identified fusions and exon-skipping events (FGFR3, BRAF, RET) are potentially treatable by clinical trials or early access, off-label, or compassionate-use programs. Excluding RNA NGS from the standard EGFR TKI resistance workup completely will therefore result in missing potentially treatable resistance mechanisms in 4% of patients in the early TKI group and 10% of osimertinib patients, 8% overall. This percentage may be even higher in patients treated with first-line osimertinib because fusions have been found to be more prevalent in that group.16

### Challenge #3 Amplification Detection

In this study, we screened for relevant amplifications with MET ISH and HER2 ISH and IHC, including DNA NGS copy number variation. MET ISH was performed in 282 cases, 88% of all cases overall. In 22 cases (7%), there was not enough tissue to complete the analysis, and in four cases (1%), the result was invalid. In the remaining 256 cases, MET amplification was identified in six cases (5%) in the early TKI group and in 17 cases (12%) in the osimertinib group. HER2 ISH was performed in 196 cases overall (62%). In 11 cases (3%), there was not enough tissue available and twice the ISH result was invalid (1%). In the 183 other cases, HER2 amplification was identified six times (8% of successful analyses) in the early TKI group and five times (5% of successful analyses) in the osimertinib group. ISH results for both MET and HER2 are summarized in Table 2.

**Table 2:** MET and HER2 ISH Results Per TKI Treatment Group

MET ISH	Early TKI Group (n = 114), n (%)	Osimertinib Group (n = 142), n (%)	*p* Value
Not amplified	108 (95)	125 (88)	0.12
Low amplification (6–10 copies)	1 (1)	2 (1)	
High amplification (>10 copies)	4 (4)	11 (8)	
High amplification in part of the tumor cells (clonal heterogeneity)	1 (1)	4 (3)	
HER2 ISH	Early TKI group (n = 76), n (%)	Osimertinib group (n = 110), n (%)	
Not amplified	70 (92)	102 (95)	0.53
Low amplification (6–10 copies)	1 (1)	3 (3)	
High amplification (>10 copies)	5 (7)	2 (2)	
High amplification in part of the tumor cells (clonal heterogeneity)	0	0	

*Note: p* values are calculated by pooling all amplified cases and performing Fisher’s exact test. Cases in which MET or HER2 ISH was not performed or was unsuccessful were not included in this table.

ISH, in situ hybridization; TKI, tyrosine kinase inhibitor.

**Figure 5:**
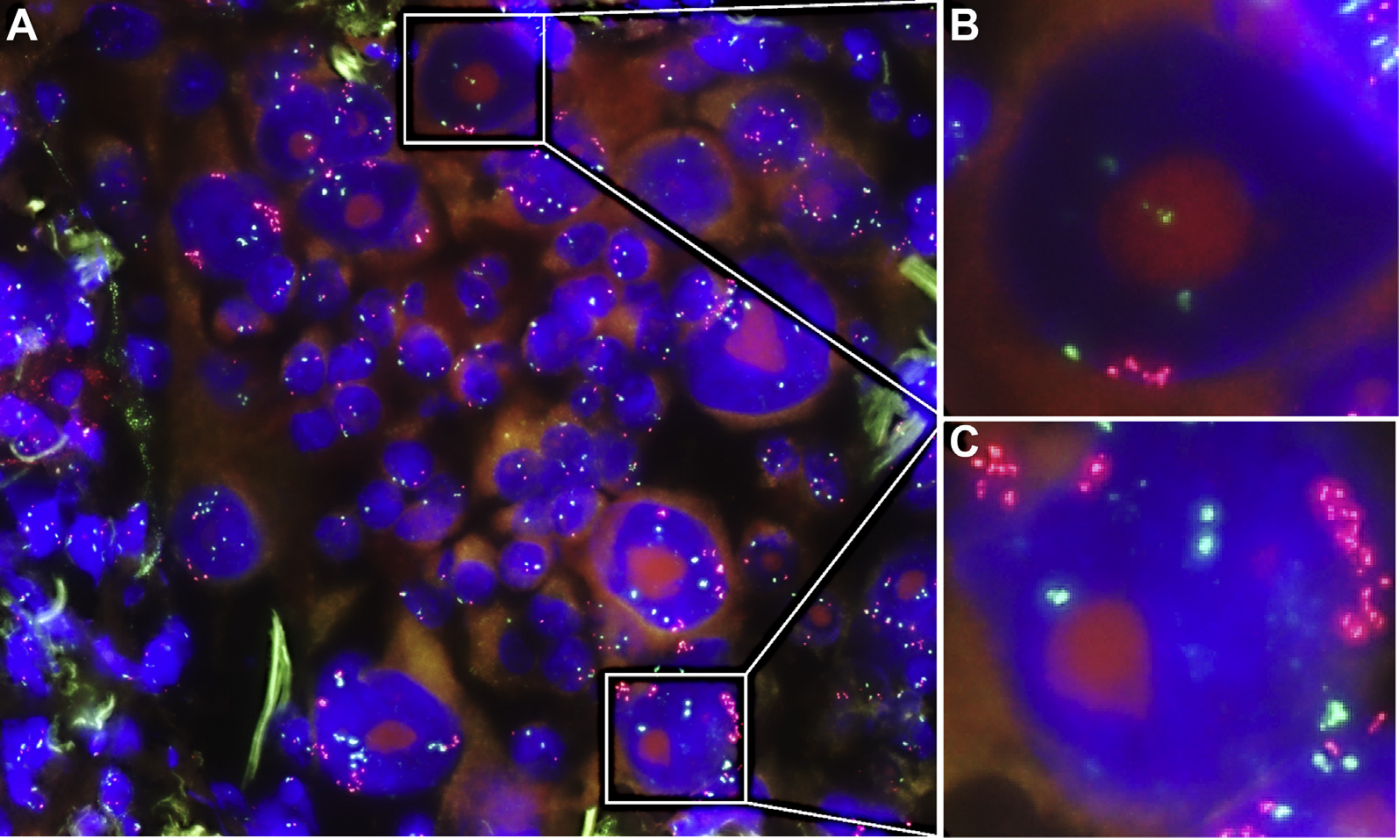
MET amplification ISH, cytology specimen. Red dots: MET probes; green dots: centromere 7 probes. In several tumor cells, the MET:centromere 7 ratio is greater than 10, but in other tumor cells, this ratio is 1. Overall, the MET-amplified tumor cells were a minority in this slide (approximately 25% of tumor cells), and the MET amplification was therefore not detected with NGS. (*A*) ISH overview. (*B*) Tumor cell without MET amplification, close-up. (*C*) Tumor cell with high (>10 copies) MET amplification, close-up. ISH, in situ hybridization; NGS, next-generation sequencing.

Most MET and HER2 amplifications were identified with both ISH and DNA NGS. Nevertheless, several amplifications were exclusively detected with ISH. The results from cases that underwent both DNA NGS and ISH are outlined in Table 3. In the three cases in which DNA NGS detected copy number variation for MET, but ISH reported no amplifications, these were all due to polysomy, which was described in the MET ISH report. In the eight cases in which MET amplification was detected by ISH but missed in DNA NGS, this was due to one of the following four reasons: (1) the amplification was present only in part of the tumor cells (an example of which is provided in Fig. 5*A–C*) in three cases; (2) low tumor cell percentage or low DNA input in three cases; (3) low amplification (5–10 copies) in one case; and (4) decreased accuracy of the copy number analysis owing to a very high amplification in another gene in one case. Omitting MET ISH from the EGFR resistance workup would therefore have resulted in misdiagnosing (missing and overdiagnosing) MET amplifications in 4% of resistance biopsies.

**Table 3:** ISH and DNA NGS Copy Number Analysis Comparison

MET ISH	DNA NGS: No Amplification	DNA NGS: Amplification
No amplification	224	3
Amplification	8	13
HER2 ISH	DNA NGS: no amplification	DNA NGS: amplification
No amplification	167	0
Amplification	2	7

ISH, in situ hybridization; NGS, next-generation sequencing.

For HER2, all amplifications detected with DNA NGS were also detected with ISH, but two amplifications (22%) were exclusively found by ISH. In both cases, a low amplification (6–10 copies) was identified in ISH, which was missed in DNA NGS, even though the tumor cell percentage was adequate (50% and 80%). Omitting HER2 ISH from the EGFR resistance workup would therefore have resulted in missing HER2 amplification in 1% of the cases, which constitutes 22% of all HER2 amplifications.

Several amplifications, both in HER2 and MET, were exclusively identified by ISH, usually owing to low tumor cell percentage, low amplification (fewer copies), or the amplification being present only in part of the tumor cells (clonal heterogeneity). Only cases with sufficient tissue for DNA NGS and MET ISH were included.

HER2 IHC and HER2 ISH were both performed in 180 cases. The results for these cases are outlined in Table 4. All cases in which ISH identified an amplification had high HER2 expression (3+). Omitting either HER2 ISH or HER2 IHC therefore would not have resulted in misdiagnosing any HER2 amplifications.

**Table 4:** HER2 ISH Versus HER2 IHC

HER2 ISH	IHC: 0	IHC: 1+	IHC: 2+	IHC: 3+
ISH: 0–5 copies	105	52	13	0
ISH: 6–10 copies	0	0	0	4
ISH: >10 copies	0	0	0	6

ISH, in situ hybridization; IHC, immunohistochemistry.

### Challenge #4 Tissue Scarcity

In DNA NGS, 12 cases were of insufficient quality for a complete analysis (4%). This was true for 24 cases (18%) of all attempted RNA NGS analyses. MET ISH was not possible in 26 cases (9% of all attempts); for HER2 ISH, this was 13 cases (7% of all attempted HER2 ISH); and for HER2 IHC, seven cases (3% of all attempts). This is a relatively low dropout, compared with the results from hybrid capture NSCLC studies in literature33 or WGS. There was no clear correlation between dropout and specimen type or biopsy site.

### Challenge #5 Comparison With Pretreatment Biopsy

All resistance biopsies underwent morphologic examination by pulmonary pathologists. In several cases, transformation to another morphologic phenotype was observed. In the early TKI group, small-cell transformation was observed twice (1%) and squamous transformation once (1%). In the osimertinib group, five cases transformed to a small-cell phenotype (3%) and two to a squamous phenotype (1%).

In 28 cases (9%), molecular comparison to the pretreatment biopsy was not optimal. This was often due to the use of small (circulating tumor)DNA NGS panels on the pretreatment biopsy, which do not cover amplifications and fusions. In this setting, it is difficult to determine which molecular alterations were novel compared with the pretreatment biopsy, especially in second-line osimertinib cases. These 28 cases were therefore excluded from the mutation prevalence analyses in this study.

### Loss of T790M After Osimertinib

A total of 84 cases harbored a T790M mutation on start of osimertinib treatment. In 47 of those cases, the T790M mutation was not identified anymore in the post-osimertinib resistance biopsy (54%). The T790M mutation was lost significantly more often (*p* = 0.045) in cases without a new resistance mechanism, as illustrated in Table 5.

**Table 5:** Loss of T790M and Detection of New Resistance Mechanisms

Characteristics	Loss of T790M	T790M Not Lost	*p* Value
No resistance mechanism	25	11	0.045^a^
New resistance mechanism	22	26	
Treatment time	452	595	0.07^b^
Age	66	59	0.002^b^
Smoking pack-years	5.5	3.5	0.43^b^
Never smoker	27	28	0.11^a^
Ever smoker	20	9	

a *p* values were calculated with Fisher’s exact test.

b *p* values were calculated with independent *t* test.

### Acquired Driver Mutations

In 110 cases (36% of successful analyses), new driver mutations (which were not present in the pre-TKI biopsy) were discovered in the resistance biopsy, whereas in 14 cases (5%), a previously present driver mutation was not identified anymore. The meaning of this remains unknown. Patients with new driver mutations were not different in age, treatment time, smoking status, pack-years, or TKI treatment group.

## Discussion

In this study, we analyzed the molecular findings of 320 biopsy specimens submitted for EGFR TKI resistance in three different hospitals. Acquired resistance mechanisms were identified in 54% of all cases by DNA NGS, RNA NGS, MET ISH, HER2 ISH, and HER2 IHC. Each additional molecular test had a substantial yield: omitting RNA NGS would lead to misdiagnosis in 8% of cases, MET ISH in 4%, and HER2 ISH and IHC in 1%.

By comparing the results from these assays, we illustrated how clonal heterogeneity can decrease the sensitivity of DNA NGS, especially for amplifications and in cases with a low tumor cell percentage. We revealed that clonal heterogeneity frequently occurs in EGFR TKI-resistant NSCLC, and that it may lead to problematic discrepancies between DNA NGS and ISH. Furthermore, we proved that acquired resistance mechanisms for EGFR TKIs are not always mutually exclusive, both in the early TKI group (co-occurring mechanisms in 9%) and in the osimertinib group (co-occurring mechanisms in 6%).

**Figure 6:**
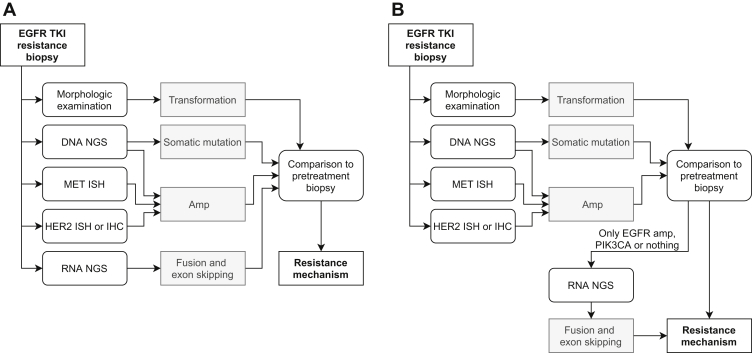
Summary of recommendations for EGFR TKI resistance screening. (*A*) Parallel approach, safest option. (*B*) Sequential approach, preferred when limited tissue or financial feasibility is an issue. amp, amplification; IHC, immunohistochemistry; ISH, in situ hybridization; NGS, next-generation sequencing; TKI, tyrosine kinase inhibitor.

Owing to clonal heterogeneity and the co-occurrence of acquired resistance mechanisms, performing a parallel workup that includes DNA NGS, RNA NGS, MET ISH, and HER2 ISH or IHC is the most sensitive and most comprehensive option for molecular diagnostics in the setting of a routine EGFR TKI resistance biopsy (Fig. 6*A*). Nevertheless, the added benefit of RNA NGS is limited for cases in which a “strong” resistance mechanism has already been identified with DNA NGS, MET ISH, and HER2 ISH or IHC: in this study, 0 case harbored an additional fusion or exon-skipping event, and observations in the literature are limited. In practice, however, there are several arguments that favor a parallel approach. First, with different types of tissue (FFPE blocks, cytology smears, cytology blocks, and combinations thereof) that are presented, logistics are challenging not only for requesting pathologists but also for the laboratory. Second, a parallel workup is tissue efficient, and third, when the tumor progresses, you can compare results of the analysis of that biopsy with a full analysis. Nevertheless, if substantial concerns exist with regard to tissue exhaustion (when DNA and RNA are isolated in separate steps), financial feasibility, or lack of capacity to perform the tests, it is justifiable to opt for a sequential approach, in which DNA NGS, MET ISH, HER2 ISH, or IHC is performed, and additional RNA NGS is performed in case no resistance mechanisms or only PIK3CA or EGFR amplification is identified (Fig. 6*B*). Nevertheless, it should be noted that this sequential approach takes longer, which can be problematic for patients, and the risk of missing relevant fusions—however small—is likely not 0%, as resistance mechanisms may co-occur.

Either HER2 ISH or IHC can be used; they are equally accurate for detecting HER2 amplifications. The dropout of this approach is relatively low, especially compared with large hybrid capture panels^33^ and WGS, which might become the preferred method in the future, when technological advancements reduce the dropout rates, which are especially high when using small biopsies and cytology material. The low dropout rate in this study is in part due to the isolation method: isolating total nucleic acid and splitting in RNA and DNA later is a meaningful step in the EGFR resistance workup, as described.^32^

A potential limitation of this study, owing to the retrospective and “real world” nature, is that most patients in the osimertinib group were treated with osimertinib as a second, third, or even fourth treatment line. Nevertheless, because osimertinib is now approved for first-line treatment, most patients in the future will present with first-line osimertinib resistance. Literature suggests that the mutations found in first-line osimertinib resistance are comparable with those in second-line osimertinib resistance, but with more fusions and exon-skipping events.^20^ If that is true, our recommendations for a diagnostic sequence will still be applicable, and the yield of RNA NGS will even be higher. In addition, owing to the “real world” nature of our research, our cohort is different from previously described registration-trial cohorts with regard to inclusion criteria and resistance mechanism prevalence.^4^^,^^34^

Another potential limitation is the variation between laboratories. Although each laboratory in this study had a similar NEN-EN-ISO 15189 accreditation and approach, and panels overlapped substantially, there might still have been subtle differences. We believe that a more uniform approach could benefit future patients with cancer and streamline communication between laboratories.

Owing to the retrospective nature of this study and currently lacking of robust recommendations for molecular diagnostics after EGFR TKI resistance, not all molecular tests were performed for all cases in this study. Especially the number of cases tested for RNA NGS was limited.

Another caveat is the clinical benefit of screening for acquired resistance mechanisms after EGFR TKI resistance. Robust proof that screening for these mutations actually improves survival is still lacking. Nevertheless, owing to the rapidly changing landscape of targeted treatment options and swift accessibility by trials, compassionate-use, and early access programs, we assume that screening for these acquired resistance mechanisms will become an important requirement. In our cohort, patients were frequently included in a clinical trial when a resistance mechanism was identified.

Many biopsy specimens in this study revealed a loss of T790M or had acquired a new driver mutation during the TKI treatment. The clinical consequences of these findings are unknown and should be investigated further. Ultimately, we like to discover whether this is a sign of tumor dedifferentiation or therapy-induced selection and has any (progression-free) survival consequences.

The aim of this study was to recommend the most optimal molecular diagnostic sequence for the EGFR TKI resistance setting. In 54% of all EGFR resistance biopsies, we were able to identify a resistance mechanism with our molecular diagnostics sequence. Although mechanisms of acquired resistance might be discovered in the future, our approach (combining DNA NGS, RNA NGS, MET ISH, HER2 ISH, or HER2 IHC) is currently the most comprehensive and safest option for patients with acquired resistance to EGFR TKIs.

## CRediT Authorship Contribution Statement

**Liesbeth M.Hondelink:** Conceptualization, Methodology, Formal analysis, Investigation, Data curation, Writing - original draft, Writing - review & editing, Visualization.

**MerelJebbink:** Conceptualization, Investigation, Writing - review & editing.

**Jan H.von der Thüsen,Tomvan Wezel, KimMonkhorst:** Conceptualization, Methodology, Investigation, Resources, Data curation, Writing - original draft, Writing - review & editing, Supervision, Project administration.

**DanielleCohen:** Conceptualization, Methodology, Investigation, Resources, Data curation, Writing - original draft, Writing - review & editing, Visualization, Supervision, Project administration.

**HendrikusJ. Dubbink,Mirjam C.Boelens:** Conceptualization, Resources, Data curation, Writing - review & editing.

**Marthe S.Paats, A. Dingemans,Adrianus J.de Langen,EgbertF. Smit,Pieter E.Postmus:** Conceptualization, Resources, Writing - review & editing.

## Supplementary Data

Supplementary Methods

Supplementary Figure 1 Patient journey. In the real world clinical setting, patients are often treated with multiple lines of TKIs and screened for resistance mechanisms several times. Each resistance biopsy was included separately in our cohort.
