# Supplementary material for: Real-World Approach for Molecular Analysis of Acquired EGFR Tyrosine Kinase Inhibitor Resistance Mechanisms in NSCLC
Source: JTO Clin Res Rep. 2021 Nov 1;2(12):100252. doi: 10.1016/j.jtocrr.2021.100252 (PMC8608608; doi:10.1016/j.jtocrr.2021.100252)
Supplement: Supplementary Methods [file mmc1.docx]

**Supplementary Methods**

HER2 immunohistochemistry (IHC):

- Leiden University Medical Center (LUMC): 1- to 10-μm thick slides were cut using a Leica RM2255 Automated Microtome. Staining was performed with Dako A0485 antibody with Dako Omnis immunostainer and Dako EnVision Flex+ in a lab-developed test with citrate and a 1:100 dilution.
- Erasmus Medical Center (EMC): HER2 immunohistochemistry was not performed.
- The Netherlands Cancer Institute (NKI): Benchmark Ultra with Ventana 4B5 antibody.

MET and HER2 in situ hybridization (ISH):

- LUMC HER2: HER2 Dual ISH Ventana Benchmark Ultra.
- LUMC MET: MET Leica/Kreatech Dual Color MET/CEN7 probe.
- EMC HER2: Ventana HER2 Dual ISH DNA Probe Cocktail assay on Benchmark Ultra.
- EMC MET: Zytolight Spec MET/CEN7 dual color probe.
- NKI HER2: HER2 dual SISH Roche Diagnostics.
- NKI MET: MET dual break apart MET/CEN7 probe Roche Diagnostics.

DNA-based targeted next generation sequencing (DNA NGS)

- LUMC: customized Cancer Hotspot Panel, covering hotspots in ABL1, AKT1, ALK, APC, ARAF, ATM, BRAF, CARD11, CD79A, CD79B, CDH1, CDK4, CDKN2A, CIC, CSF1R, CTNNB1, EGFR, EIF1AX, ERBB2, ERBB3, ERBB4, EZH2, FAK (PTK2), FBXW7, FGFR1, FGFR2, FGFR3, FLT3, FOXL2, GNA11, GNAQ, GNAS, H3F3A, H3F3B, HNF1A, HRAS, IDH1, IDH2, JAK2, JAK3, KDR, KIT, KRAS, MAP2K1, MAP2K2, MAP2K4, MAP3K1, MDM2, MED12, MET, MLH1, MPL, MUTYH, MYC, MYD88, NOTCH1, NPM1, NRAS, PDGFRA, PDGFRB, PIK3CA, POLE, PTEN, PTPN11, RB1, RET, SMAD4, SMARCB1, SMO, SRC, STK11, TP53, and VHL. Raw data was mapped against the reference genome (GRCh37/hg19) using the TMAP 5.0.7 software (<https://github.com/iontorrent/TS>), variant calling was performed with Ion Torrent specific caller, Torrent Variant Caller (TVC)-5.0.2, using Variant Caller Parameter for Cancer Hotspot Panel version 2. Variant interpretation was performed with Genetic Assistant (<http://softgenetics.com/GeneticistAssistant_2.php>). Copy number analysis, visualization of results, loss of heterozygosity, and chromosomal imbalances were preformed using the Next-Generation Sequencing Expert shiny app (<https://git.lumc.nl/druano/NGSE>).
- EMC: IonTorrent custom targeted next generation sequencing panel version 5.1, including genes: CDKN2A (coverage 98%), PTEN (coverage 94%), TP53 (coverage 100%) Mutation hotspots: AKT1 (exon 3), ALK (20, 22-25), APC (14), ARAF (7), BRAF (11, 15), CTNNB1 (3, 7, 8), EGFR (18-21), HER2 (19-21), EZH2 (16), FBWX7 (9, 10), FGFR1 (4, 7, 12), FGFR2 (7, 9, 12), FGFR3 (7, 9), FOXL2 (1), GNA11 (4, 5), GNAQ (4, 5), GNAS (8, 9), HRAS (2-4), IDH1 (4), IDH2 (4), KIT (8, 9, 11, 13, 14, 17), KRAS (2-4), MAP2K1 (2, 3), MET (2, 14, 19), MYD88 (5), NOTCH1 (26, 27), NRAS (2-4), PDGFRA (12, 14, 18), PIK3CA (10, 21), POLD1 (12), POLE (9, 13), RAF1 (7), RET (11, 16), RNF43 (3, 4, 9), ROS1 (38, 41), SMAD4 (3, 9, 12), STK11 (4, 5, 8), TERT promotor. Copy number calling was performed using SeqNext, SNPittyor IonReporter.
- NKI: Illumina Ampliseq™ Cancer Hotspot Panel v2-SOCv1 covering hotspots in ABL1, AKT1, ALK, APC, ATM, BRAF, CDH1, CDKN2A, CSF1R, CTNNB1, EGFR, ERBB2, ERBB4, EZH2, FBXW7, FGFR1, FGFR2, FGFR3, FLT3, GNA11, GNAQ, GNAS, HNF1A, HRAS, IDH1, IDH2, JAK2, JAK3, KDR, KIT, KRAS, MET, MLH1, MPL, NOTCH1, NPM1, NRAS, PDGFRA, PIK3CA, POLE, PTEN, PTPN11, RB1, RET, ROS1, SMAD4, SMARCB1, SMO, SRC, STK11, TP53 and VHL. The reads were analyzed with NGS/MiSeq analysis on IonTorrent platform, copy number analysis was performed with a custom pipeline, based on CNVkit (<https://cnvkit.readthedocs.io/en/stable/>).

RNA-based targeted next generation sequencing (RNA NGS):

- LUMC: Archer FusionPlex Comprehensive Thyroid and Lung panel was used to detect fusions and exon skipping events in ALK, AXL, BRAF, CCND1, EGFR, FGFR1, FGFR2, FGFR3, MET, NTRK1, NTRK2, NTRK3, NRG1, PPARG, RAF1, RET, ROS1 and THADA, as well as somatic mutations in ALK, AKT1, BRAF, CTNNB1, DDR2, EGFR, ERBB2, FGFR1, GNAS, HRAS, IDH1, IDH2, KRAS, MAP2K1, NRAS, NTRK1, PIK3CA, RET and ROS1 and imbalances in ALK, NTRK1, NTRK2, NTRK3, RET and ROS1. The reads were analyzed with the Comprehensive Thyroid and Lung Target Region File on IonTorrent platform and the vendor supplied software (Archer Analysis, version 5.1.7).
- EMC: Archer FusionPlex Comprehensive Thyroid and Lung panel was used to detect fusions and exon skipping events in ALK, AXL, BRAF, CCND1, EGFR, FGFR1, FGFR2, FGFR3, MET, NTRK1, NTRK2, NTRK3, NRG1, PPARG, RAF1, RET, ROS1 and THADA, as well as somatic mutations in ALK, AKT1, BRAF, CTNNB1, DDR2, EGFR, ERBB2, FGFR1, GNAS, HRAS, IDH1, IDH2, KRAS, MAP2K1, NRAS, NTRK1, PIK3CA, RET and ROS1 and imbalances in ALK, NTRK1, NTRK2, NTRK3, RET and ROS1. The reads were analyzed with the Comprehensive Thyroid and Lung Target Region File on IonTorrent platform and the vendor supplied software (Archer Analysis, version 5.1.7).
- NKI: Archer FusionPlex Lung panel was used to detect fusions and exon skipping events in ALK, BRAF, EGFR, FGFR1, FGFR2, FGFR3, MET, NRG1, NTRK1, NTRK2, NTRK3, RET and ROS1, as well as somatic mutations and imbalances in ALK, BRAF, EGFR, KRAS, RET and ROS1. The reads were analyzed with the Lung Target Region File on IonTorrent platform and the vendor supplied software (Archer Analysis, version 5.1.7).

DNA/RNA isolation methods:

- LUMC: tissue was obtained with microdissection or punching. Five 10 μm slides were used for isolating total nucleic acid using a tissue preparation system robot from Siemens, as was previously described. [10] The same total nucleic acid sample was used for both DNA and RNA NGS. When the tissue block did not contain sufficient tumor or was inavailable, tissue was scraped from cytology or H&E slides. The nucleic acid solution was stored at -20°C short-term and at -70°C long-term.
- EMC: tissue was obtained with microdissection or punching. RNA and DNA were obtained separately. DNA was isolated with Chelex or Maxwell, as described in previous literature. [11] RNA is isolated with Qiagen method. DNA is stored at -20°C and RNA at -80°C.
- NKI: DNA and RNA were isolated separately with Qiagen FFPE prep kit. DNA was stored at -20°C and RNA at -80°C for future use.
