# Supplementary figures and images for: Real-World Approach for Molecular Analysis of Acquired EGFR Tyrosine Kinase Inhibitor Resistance Mechanisms in NSCLC
Source: JTO Clin Res Rep. 2021 Nov 1;2(12):100252. doi: 10.1016/j.jtocrr.2021.100252 (PMC8608608; doi:10.1016/j.jtocrr.2021.100252)

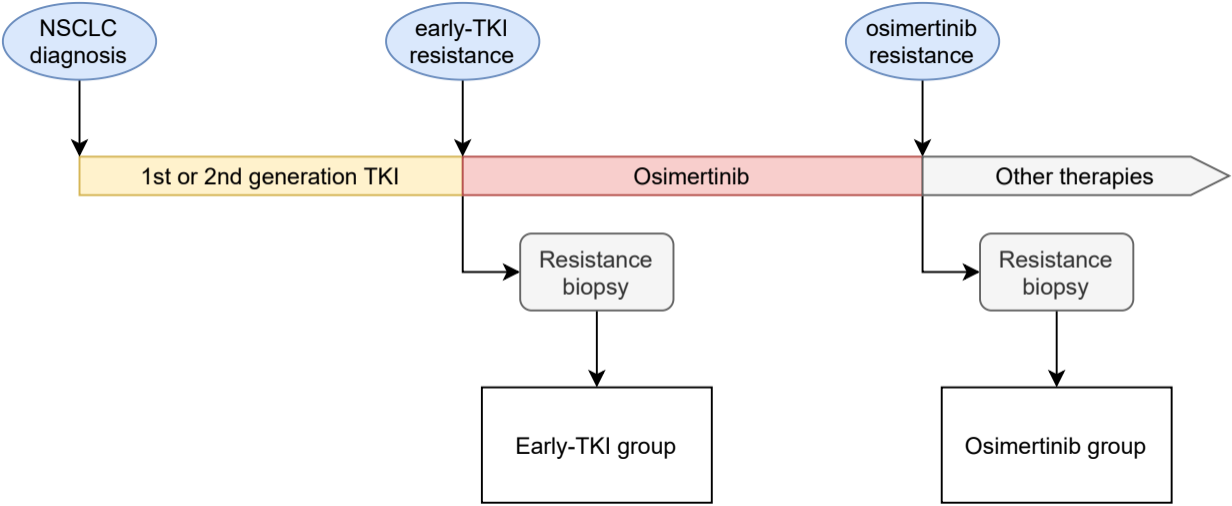

Supplement: Supplementary Figure 1 — Patient journey. In the real world clinical setting, patients are often treated with multiple lines of TKIs and screened for resistance mechanisms several times. Each resistance biopsy was included separately in our cohort. [file mmc2.pdf]
