# Supplementary material for: Real-World Approach for Molecular Analysis of Acquired EGFR Tyrosine Kinase Inhibitor Resistance Mechanisms in NSCLC
Source: JTO Clin Res Rep. 2021 Nov 1;2(12):100252. doi: 10.1016/j.jtocrr.2021.100252 (PMC8608608; doi:10.1016/j.jtocrr.2021.100252)
Supplement: Supplementary_Table_1 [file mmc3.docx]

Supplementary Table 1

|  | **Early-TKI group** | **Osimertinib group** | **P-value** |
| --- | --- | --- | --- |
| PD-L1 expression |  |  | 0.14 |
| PD-L1 negative (<1%) | 23 (36%) | 42 (42%) |  |
| PD-L1 weak-positive (1-50%) | 17 (27%) | 34 (34%) |  |
| PD-L1 strong-positive (50-100%) | 24 (38%) | 23 (23%) |  |
|  |  |  |  |
| Smoking status |  |  | 0.00003^c^ |
| Never-smoker | 69 (43%) | 100 (63%) |  |
| Former smoker | 44 (27%) | 38 (24%) |  |
| Current smoker | 12 (7%) | 13 (8%) |  |
| Unknown | 36 (22%) | 8 (5%) |  |
|  |  |  |  |
| Packyears | 6.1 (0 – 50) | 5.1 (0 – 90) | 0.53^b^ |
